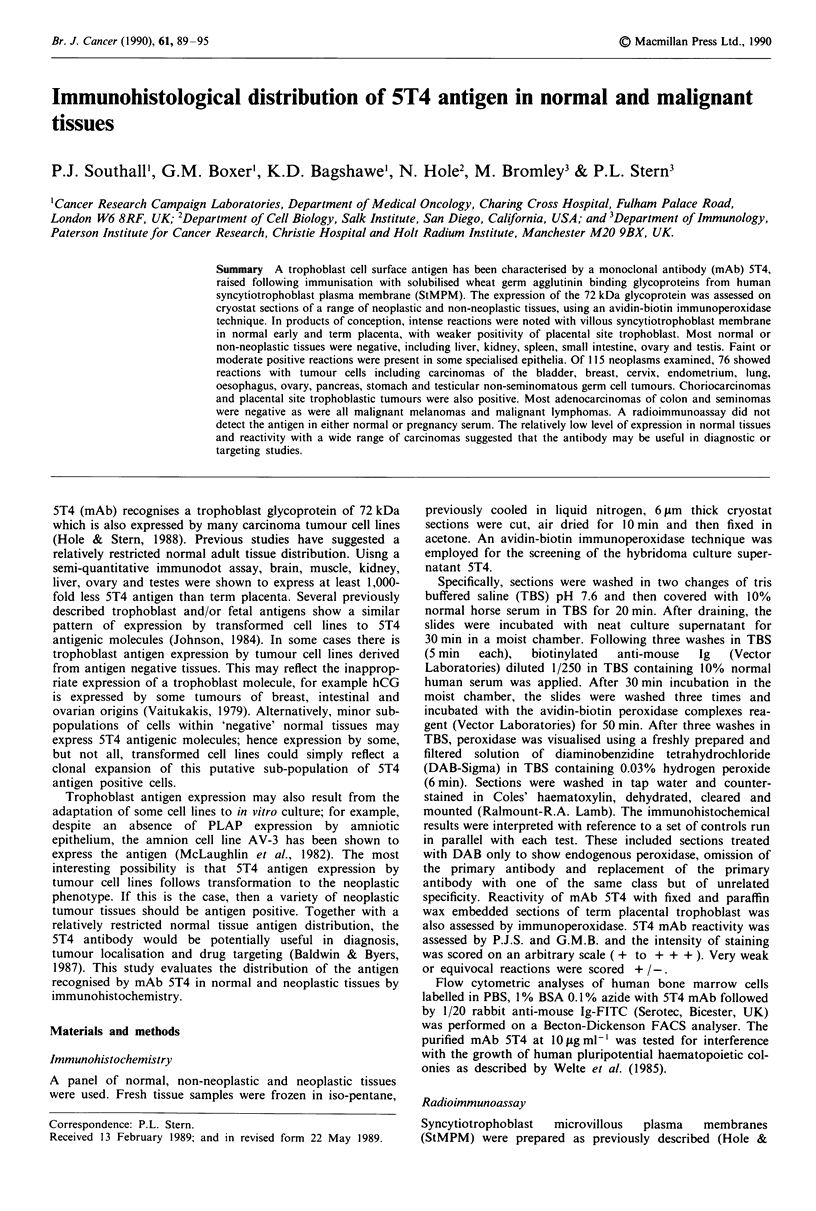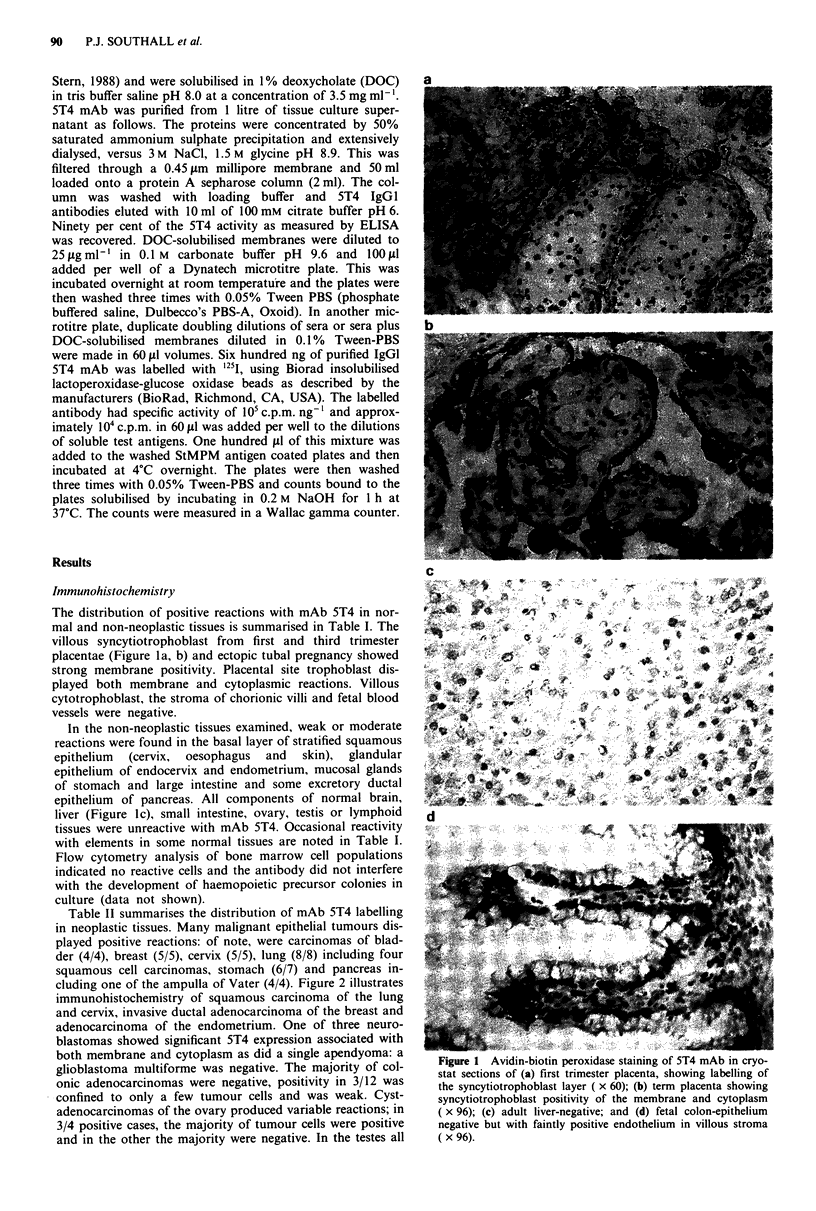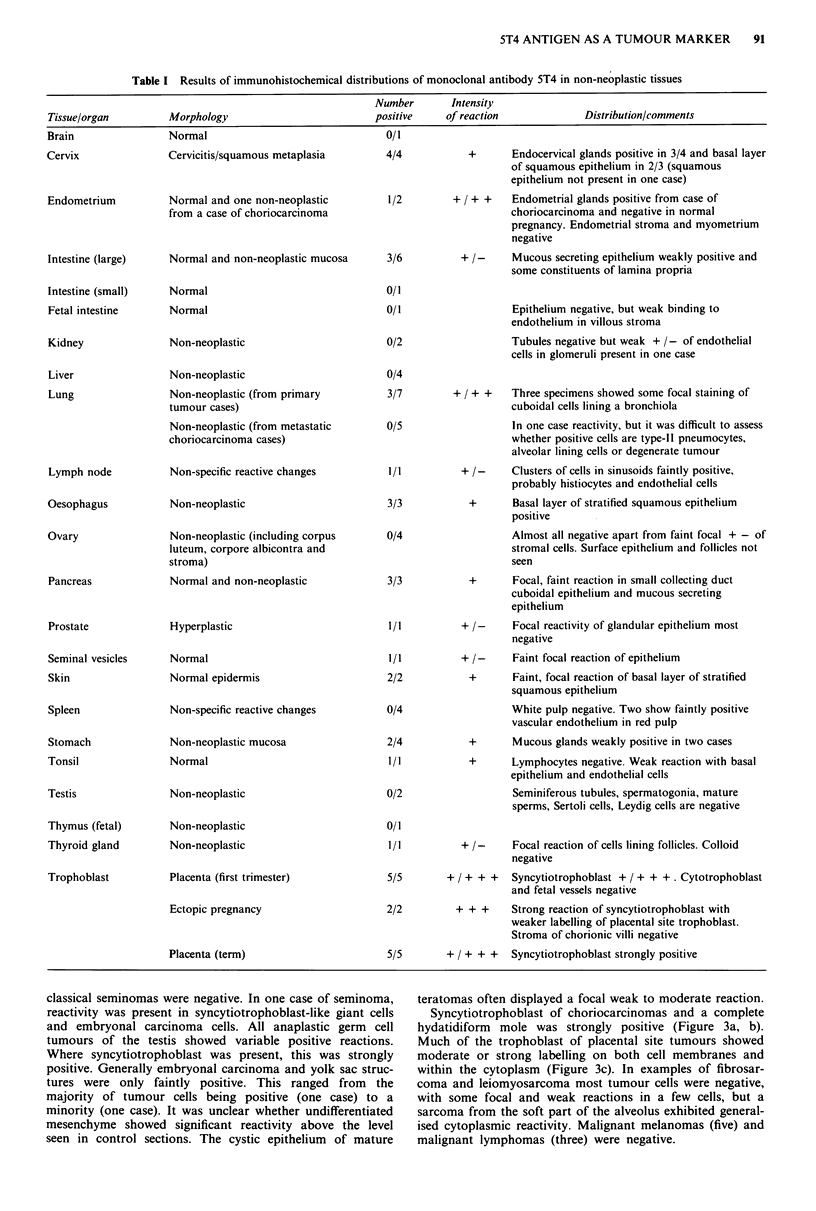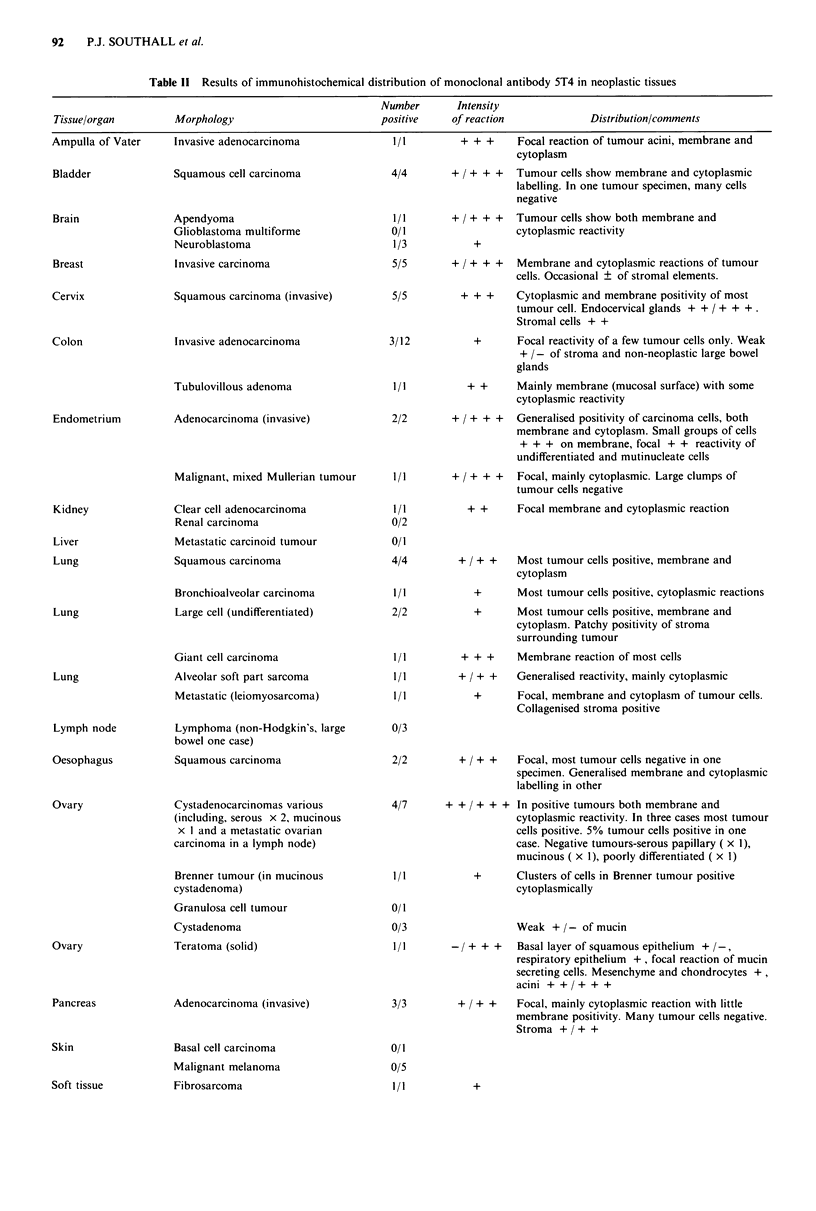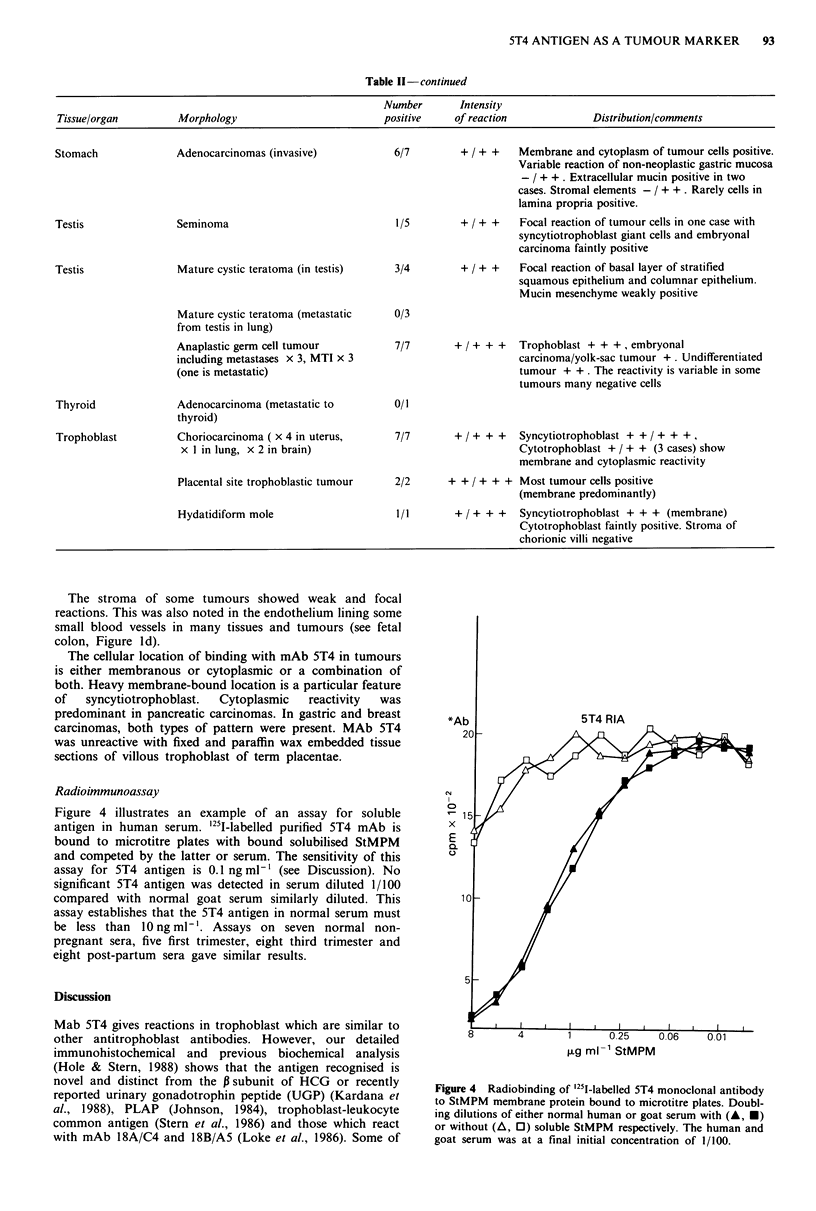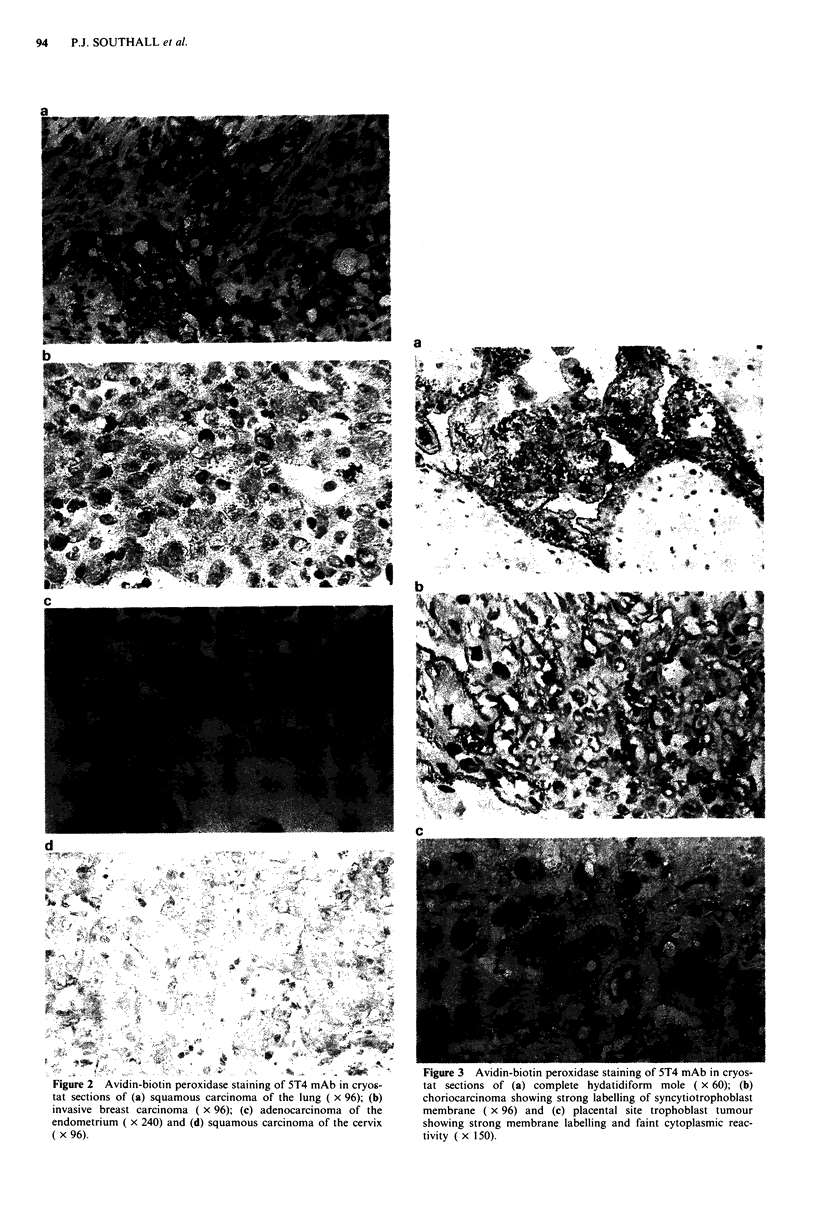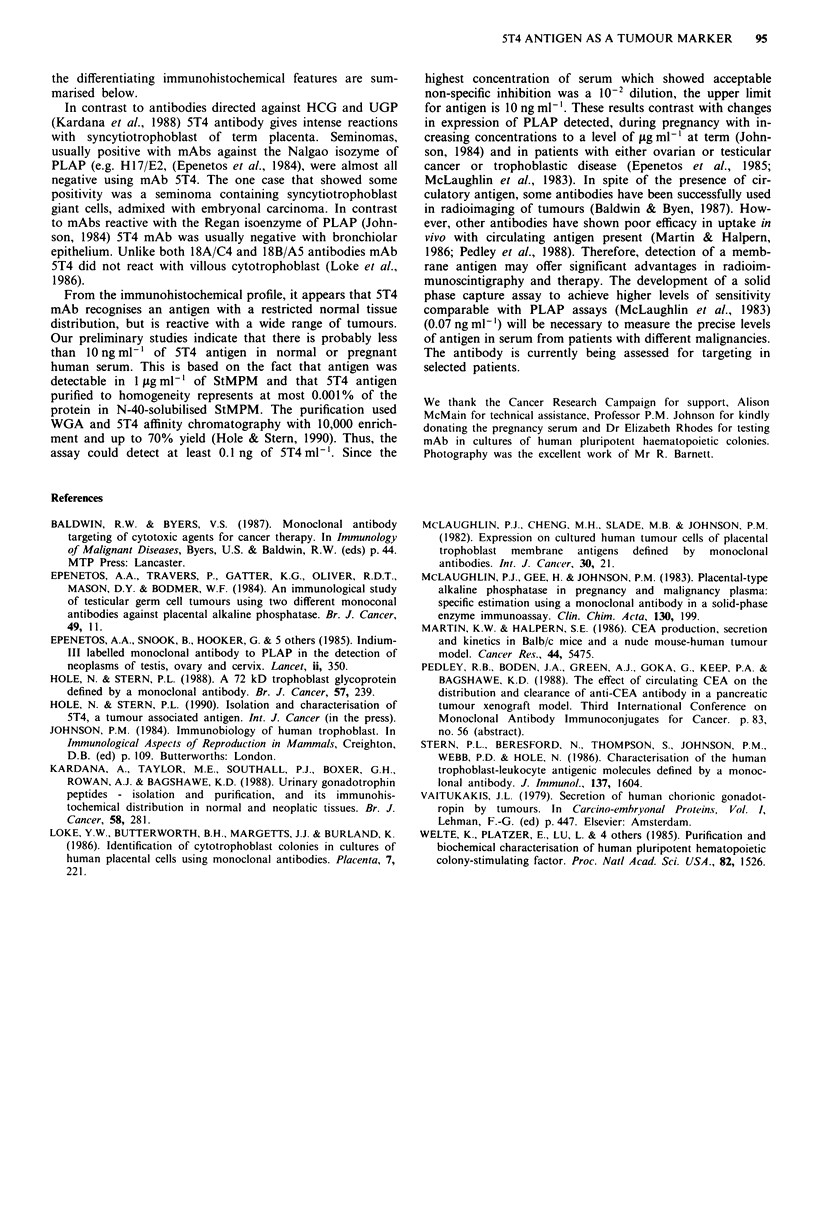# Immunohistological distribution of 5T4 antigen in normal and malignant tissues.

**DOI:** 10.1038/bjc.1990.20

**Published:** 1990-01

**Authors:** P. J. Southall, G. M. Boxer, K. D. Bagshawe, N. Hole, M. Bromley, P. L. Stern

**Affiliations:** Department of Medical Oncology, Charing Cross Hospital, London, UK.

## Abstract

**Images:**


Br. J. Cancer (1990), 61, 89–95 © Macmillan Press Ltd., 1990

### Immunohistological distribution of 5T4 antigen in normal and malignant tissues

P.J. Southall[1], G.M. Boxer[1], K.D. Bagshawe[1], N. Hole[2], M. Bromley[3] & P.L. Stern[3]

[1]*Cancer Research Campaign Laboratories, Department of Medical Oncology, Charing Cross Hospital, Fulham Palace Road, London W6 8RF, UK;* [2]*Department of Cell Biology, Salk Institute, San Diego, California, USA; and* [3]*Department of Immunology, Paterson Institute for Cancer Research, Christie Hospital and Holt Radium Institute, Manchester M20 9BX, UK.*

**Summary** A trophoblast cell surface antigen has been characterised by a monoclonal antibody (mAb) 5T4, raised following immunisation with solubilised wheat germ agglutinin binding glycoproteins from human syncytiotrophoblast plasma membrane (StMPM). The expression of the 72 kDa glycoprotein was assessed on cryostat sections of a range of neoplastic and non-neoplastic tissues, using an avidin-biotin immunoperoxidase technique. In products of conception, intense reactions were noted with villous syncytiotrophoblast membrane in normal early and term placenta, with weaker positivity of placental site trophoblast. Most normal or non-neoplastic tissues were negative, including liver, kidney, spleen, small intestine, ovary and testis. Faint or moderate positive reactions were present in some specialised epithelia. Of 115 neoplasms examined, 76 showed reactions with tumour cells including carcinomas of the bladder, breast, cervix, endometrium, lung, oesophagus, ovary, pancreas, stomach and testicular non-seminomatous germ cell tumours. Choriocarcinomas and placental site trophoblastic tumours were also positive. Most adenocarcinomas of colon and seminomas were negative as were all malignant melanomas and malignant lymphomas. A radioimmunoassay did not detect the antigen in either normal or pregnancy serum. The relatively low level of expression in normal tissues and reactivity with a wide range of carcinomas suggested that the antibody may be useful in diagnostic or targeting studies.

5T4 (mAb) recognises a trophoblast glycoprotein of 72 kDa which is also expressed by many carcinoma tumour cell lines (Hole & Stern, 1988). Previous studies have suggested a relatively restricted normal adult tissue distribution. Uisng a semi-quantitative immunodot assay, brain, muscle, kidney, liver, ovary and testes were shown to express at least 1,000-fold less 5T4 antigen than term placenta. Several previously described trophoblast and/or fetal antigens show a similar pattern of expression by transformed cell lines to 5T4 antigenic molecules (Johnson, 1984). In some cases there is trophoblast antigen expression by tumour cell lines derived from antigen negative tissues. This may reflect the inappropriate expression of a trophoblast molecule, for example hCG is expressed by some tumours of breast, intestinal and ovarian origins (Vaitukakis, 1979). Alternatively, minor sub-populations of cells within 'negative' normal tissues may express 5T4 antigenic molecules; hence expression by some, but not all, transformed cell lines could simply reflect a clonal expansion of this putative sub-population of 5T4 antigen positive cells.

Trophoblast antigen expression may also result from the adaptation of some cell lines to *in vitro* culture; for example, despite an absence of PLAP expression by amniotic epithelium, the amnion cell line AV-3 has been shown to express the antigen (McLaughlin *et al.*, 1982). The most interesting possibility is that 5T4 antigen expression by tumour cell lines follows transformation to the neoplastic phenotype. If this is the case, then a variety of neoplastic tumour tissues should be antigen positive. Together with a relatively restricted normal tissue antigen distribution, the 5T4 antibody would be potentially useful in diagnosis, tumour localisation and drug targeting (Baldwin & Byers, 1987). This study evaluates the distribution of the antigen recognised by mAb 5T4 in normal and neoplastic tissues by immunohistochemistry.

### Materials and methods

### *Immunohistochemistry*

A panel of normal, non-neoplastic and neoplastic tissues were used. Fresh tissue samples were frozen in iso-pentane, previously cooled in liquid nitrogen, 6 μm thick cryostat sections were cut, air dried for 10 min and then fixed in acetone. An avidin-biotin immunoperoxidase technique was employed for the screening of the hybridoma culture supernatant 5T4.

Specifically, sections were washed in two changes of tris buffered saline (TBS) pH 7.6 and then covered with 10% normal horse serum in TBS for 20 min. After draining, the slides were incubated with neat culture supernatant for 30 min in a moist chamber. Following three washes in TBS (5 min each), biotinylated anti-mouse Ig (Vector Laboratories) diluted 1/250 in TBS containing 10% normal human serum was applied. After 30 min incubation in the moist chamber, the slides were washed three times and incubated with the avidin-biotin peroxidase complexes reagent (Vector Laboratories) for 50 min. After three washes in TBS, peroxidase was visualised using a freshly prepared and filtered solution of diaminobenzidine tetrahydrochloride (DAB-Sigma) in TBS containing 0.03% hydrogen peroxide (6 min). Sections were washed in tap water and counterstained in Coles' haematoxylin, dehydrated, cleared and mounted (Ralmount-R.A. Lamb). The immunohistochemical results were interpreted with reference to a set of controls run in parallel with each test. These included sections treated with DAB only to show endogenous peroxidase, omission of the primary antibody and replacement of the primary antibody with one of the same class but of unrelated specificity. Reactivity of mAb 5T4 with fixed and paraffin wax embedded sections of term placental trophoblast was also assessed by immunoperoxidase. 5T4 mAb reactivity was assessed by P.J.S. and G.M.B. and the intensity of staining was scored on an arbitrary scale ( + to + + + ). Very weak or equivocal reactions were scored + / −.

Flow cytometric analyses of human bone marrow cells labelled in PBS, 1% BSA 0.1% azide with 5T4 mAb followed by 1/20 rabbit anti-mouse Ig-FITC (Serotec, Bicester, UK) was performed on a Becton-Dickenson FACS analyser. The purified mAb 5T4 at 10 $\mu g\, ml^{-1}$ was tested for interference with the growth of human pluripotential haematopoietic colonies as described by Welte *et al.* (1985).

### *Radioimmunoassay*

Syncytiotrophoblast microvillous plasma membranes (StMPM) were prepared as previously described (Hole &

Correspondence: P.L. Stern.
Received 13 February 1989; and in revised form 22 May 1989.

Stern, 1988) and were solubilised in 1% deoxycholate (DOC) in tris buffer saline pH 8.0 at a concentration of $3.5 mg ml^{-1}$. 5T4 mAb was purified from 1 litre of tissue culture supernatant as follows. The proteins were concentrated by 50% saturated ammonium sulphate precipitation and extensively dialysed, versus 3 M NaCl, 1.5 M glycine pH 8.9. This was filtered through a 0.45 μm millipore membrane and 50 ml loaded onto a protein A sepharose column (2 ml). The column was washed with loading buffer and 5T4 IgG1 antibodies eluted with 10 ml of 100 mM citrate buffer pH 6. Ninety per cent of the 5T4 activity as measured by ELISA was recovered. DOC-solubilised membranes were diluted to $25 \mu g ml^{-1}$ in 0.1 M carbonate buffer pH 9.6 and 100 μl added per well of a Dynatech microtitre plate. This was incubated overnight at room temperature and the plates were then washed three times with 0.05% Tween PBS (phosphate buffered saline, Dulbecco's PBS-A, Oxoid). In another microtitre plate, duplicate doubling dilutions of sera or sera plus DOC-solubilised membranes diluted in 0.1% Tween-PBS were made in 60 μl volumes. Six hundred ng of purified IgGl 5T4 mAb was labelled with $^{125}I$, using Biorad insolubilised lactoperoxidase-glucose oxidase beads as described by the manufacturers (BioRad, Richmond, CA, USA). The labelled antibody had specific activity of $10^5$ c.p.m. $ng^{-1}$ and approximately $10^4$ c.p.m. in 60 μl was added per well to the dilutions of soluble test antigens. One hundred μl of this mixture was added to the washed StMPM antigen coated plates and then incubated at 4°C overnight. The plates were then washed three times with 0.05% Tween-PBS and counts bound to the plates solubilised by incubating in 0.2 M NaOH for 1 h at 37°C. The counts were measured in a Wallac gamma counter.

### Results

### *Immunohistochemistry*

The distribution of positive reactions with mAb 5T4 in normal and non-neoplastic tissues is summarised in Table I. The villous syncytiotrophoblast from first and third trimester placentae (Figure 1a, b) and ectopic tubal pregnancy showed strong membrane positivity. Placental site trophoblast displayed both membrane and cytoplasmic reactions. Villous cytotrophoblast, the stroma of chorionic villi and fetal blood vessels were negative.

In the non-neoplastic tissues examined, weak or moderate reactions were found in the basal layer of stratified squamous epithelium (cervix, oesophagus and skin), glandular epithelium of endocervix and endometrium, mucosal glands of stomach and large intestine and some excretory ductal epithelium of pancreas. All components of normal brain, liver (Figure 1c), small intestine, ovary, testis or lymphoid tissues were unreactive with mAb 5T4. Occasional reactivity with elements in some normal tissues are noted in Table I. Flow cytometry analysis of bone marrow cell populations indicated no reactive cells and the antibody did not interfere with the development of haemopoietic precursor colonies in culture (data not shown).

Table II summarises the distribution of mAb 5T4 labelling in neoplastic tissues. Many malignant epithelial tumours displayed positive reactions: of note, were carcinomas of bladder (4/4), breast (5/5), cervix (5/5), lung (8/8) including four squamous cell carcinomas, stomach (6/7) and pancreas including one of the ampulla of Vater (4/4). Figure 2 illustrates immunohistochemistry of squamous carcinoma of the lung and cervix, invasive ductal adenocarcinoma of the breast and adenocarcinoma of the endometrium. One of three neuroblastomas showed significant 5T4 expression associated with both membrane and cytoplasm as did a single apendyoma: a glioblastoma multiforme was negative. The majority of colonic adenocarcinomas were negative, positivity in 3/12 was confined to only a few tumour cells and was weak. Cystadenocarcinomas of the ovary produced variable reactions; in 3/4 positive cases, the majority of tumour cells were positive and in the other the majority were negative. In the testes all a
b
c
d

**Figure 1** Avidin-biotin peroxidase staining of 5T4 mAb in cryostat sections of (**a**) first trimester placenta, showing labelling of the syncytiotrophoblast layer ( × 60); (**b**) term placenta showing syncytiotrophoblast positivity of the membrane and cytoplasm ( × 96); (**c**) adult liver-negative; and (**d**) fetal colon-epithelium negative but with faintly positive endothelium in villous stroma ( × 96).

**Table I** Results of immunohistochemical distributions of monoclonal antibody 5T4 in non-neoplastic tissues

| *Tissue/organ* | *Morphology* | *Number positive* | *Intensity of reaction* | *Distribution/comments* |
|---|---|---|---|---|
| Brain | Normal | 0/1 | | |
| Cervix | Cervicitis/squamous metaplasia | 4/4 | + | Endocervical glands positive in 3/4 and basal layer of squamous epithelium in 2/3 (squamous epithelium not present in one case) |
| Endometrium | Normal and one non-neoplastic from a case of choriocarcinoma | 1/2 | + / + + | Endometrial glands positive from case of choriocarcinoma and negative in normal pregnancy. Endometrial stroma and myometrium negative |
| Intestine (large) | Normal and non-neoplastic mucosa | 3/6 | + / − | Mucous secreting epithelium weakly positive and some constituents of lamina propria |
| Intestine (small) | Normal | 0/1 | | |
| Fetal intestine | Normal | 0/1 | | Epithelium negative, but weak binding to endothelium in villous stroma |
| Kidney | Non-neoplastic | 0/2 | | Tubules negative but weak + / − of endothelial cells in glomeruli present in one case |
| Liver | Non-neoplastic | 0/4 | | |
| Lung | Non-neoplastic (from primary tumour cases) | 3/7 | + / + + | Three specimens showed some focal staining of cuboidal cells lining a bronchiola |
| | Non-neoplastic (from metastatic choriocarcinoma cases) | 0/5 | | In one case reactivity, but it was difficult to assess whether positive cells are type-II pneumocytes, alveolar lining cells or degenerate tumour |
| Lymph node | Non-specific reactive changes | 1/1 | + / − | Clusters of cells in sinusoids faintly positive, probably histiocytes and endothelial cells |
| Oesophagus | Non-neoplastic | 3/3 | + | Basal layer of stratified squamous epithelium positive |
| Ovary | Non-neoplastic (including corpus luteum, corpore albicontra and stroma) | 0/4 | | Almost all negative apart from faint focal + − of stromal cells. Surface epithelium and follicles not seen |
| Pancreas | Normal and non-neoplastic | 3/3 | + | Focal, faint reaction in small collecting duct cuboidal epithelium and mucous secreting epithelium |
| Prostate | Hyperplastic | 1/1 | + / − | Focal reactivity of glandular epithelium most negative |
| Seminal vesicles | Normal | 1/1 | + / − | Faint focal reaction of epithelium |
| Skin | Normal epidermis | 2/2 | + | Faint, focal reaction of basal layer of stratified squamous epithelium |
| Spleen | Non-specific reactive changes | 0/4 | | White pulp negative. Two show faintly positive vascular endothelium in red pulp |
| Stomach | Non-neoplastic mucosa | 2/4 | + | Mucous glands weakly positive in two cases |
| Tonsil | Normal | 1/1 | + | Lymphocytes negative. Weak reaction with basal epithelium and endothelial cells |
| Testis | Non-neoplastic | 0/2 | | Seminiferous tubules, spermatogonia, mature sperms, Sertoli cells, Leydig cells are negative |
| Thymus (fetal) | Non-neoplastic | 0/1 | | |
| Thyroid gland | Non-neoplastic | 1/1 | + / − | Focal reaction of cells lining follicles. Colloid negative |
| Trophoblast | Placenta (first trimester) | 5/5 | + / + + + | Syncytiotrophoblast + / + + +. Cytotrophoblast and fetal vessels negative |
| | Ectopic pregnancy | 2/2 | + + + | Strong reaction of syncytiotrophoblast with weaker labelling of placental site trophoblast. Stroma of chorionic villi negative |
| | Placenta (term) | 5/5 | + / + + + | Syncytiotrophoblast strongly positive |

classical seminomas were negative. In one case of seminoma, reactivity was present in syncytiotrophoblast-like giant cells and embryonal carcinoma cells. All anaplastic germ cell tumours of the testis showed variable positive reactions. Where syncytiotrophoblast was present, this was strongly positive. Generally embryonal carcinoma and yolk sac structures were only faintly positive. This ranged from the majority of tumour cells being positive (one case) to a minority (one case). It was unclear whether undifferentiated mesenchyme showed significant reactivity above the level seen in control sections. The cystic epithelium of mature teratomas often displayed a focal weak to moderate reaction.

Syncytiotrophoblast of choriocarcinomas and a complete hydatidiform mole was strongly positive (Figure 3a, b). Much of the trophoblast of placental site tumours showed moderate or strong labelling on both cell membranes and within the cytoplasm (Figure 3c). In examples of fibrosarcoma and leiomyosarcoma most tumour cells were negative, with some focal and weak reactions in a few cells, but a sarcoma from the soft part of the alveolus exhibited generalised cytoplasmic reactivity. Malignant melanomas (five) and malignant lymphomas (three) were negative.

**Table II** Results of immunohistochemical distribution of monoclonal antibody 5T4 in neoplastic tissues

| *Tissue/organ* | *Morphology* | *Number positive* | *Intensity of reaction* | *Distribution/comments* |
|---|---|---|---|---|
| Ampulla of Vater | Invasive adenocarcinoma | 1/1 | + + + | Focal reaction of tumour acini, membrane and cytoplasm |
| Bladder | Squamous cell carcinoma | 4/4 | + / + + + | Tumour cells show membrane and cytoplasmic labelling. In one tumour specimen, many cells negative |
| Brain | Apendyoma<br>Glioblastoma multiforme<br>Neuroblastoma | 1/1<br>0/1<br>1/3 | + / + + +<br><br>+ | Tumour cells show both membrane and cytoplasmic reactivity |
| Breast | Invasive carcinoma | 5/5 | + / + + + | Membrane and cytoplasmic reactions of tumour cells. Occasional ± of stromal elements. |
| Cervix | Squamous carcinoma (invasive) | 5/5 | + + + | Cytoplasmic and membrane positivity of most tumour cell. Endocervical glands + + / + + + . Stromal cells + + |
| Colon | Invasive adenocarcinoma | 3/12 | + | Focal reactivity of a few tumour cells only. Weak + / − of stroma and non-neoplastic large bowel glands |
| | Tubulovillous adenoma | 1/1 | + + | Mainly membrane (mucosal surface) with some cytoplasmic reactivity |
| Endometrium | Adenocarcinoma (invasive) | 2/2 | + / + + + | Generalised positivity of carcinoma cells, both membrane and cytoplasm. Small groups of cells + + + on membrane, focal + + reactivity of undifferentiated and mutinucleate cells |
| | Malignant, mixed Mullerian tumour | 1/1 | + / + + + | Focal, mainly cytoplasmic. Large clumps of tumour cells negative |
| Kidney | Clear cell adenocarcinoma<br>Renal carcinoma | 1/1<br>0/2 | + + | Focal membrane and cytoplasmic reaction |
| Liver | Metastatic carcinoid tumour | 0/1 | | |
| Lung | Squamous carcinoma | 4/4 | + / + + | Most tumour cells positive, membrane and cytoplasm |
| | Bronchioalveolar carcinoma | 1/1 | + | Most tumour cells positive, cytoplasmic reactions |
| Lung | Large cell (undifferentiated) | 2/2 | + | Most tumour cells positive, membrane and cytoplasm. Patchy positivity of stroma surrounding tumour |
| | Giant cell carcinoma | 1/1 | + + + | Membrane reaction of most cells |
| Lung | Alveolar soft part sarcoma | 1/1 | + / + + | Generalised reactivity, mainly cytoplasmic |
| | Metastatic (leiomyosarcoma) | 1/1 | + | Focal, membrane and cytoplasm of tumour cells. Collagenised stroma positive |
| Lymph node | Lymphoma (non-Hodgkin's, large bowel one case) | 0/3 | | |
| Oesophagus | Squamous carcinoma | 2/2 | + / + + | Focal, most tumour cells negative in one specimen. Generalised membrane and cytoplasmic labelling in other |
| Ovary | Cystadenocarcinomas various (including, serous × 2, mucinous × 1 and a metastatic ovarian carcinoma in a lymph node) | 4/7 | + + / + + + | In positive tumours both membrane and cytoplasmic reactivity. In three cases most tumour cells positive. 5% tumour cells positive in one case. Negative tumours-serous papillary ( × 1), mucinous ( × 1), poorly differentiated ( × 1) |
| | Brenner tumour (in mucinous cystadenoma) | 1/1 | + | Clusters of cells in Brenner tumour positive cytoplasmically |
| | Granulosa cell tumour | 0/1 | | |
| | Cystadenoma | 0/3 | | Weak + / − of mucin |
| Ovary | Teratoma (solid) | 1/1 | − / + + + | Basal layer of squamous epithelium + / −, respiratory epithelium + , focal reaction of mucin secreting cells. Mesenchyme and chondrocytes + , acini + + / + + + |
| Pancreas | Adenocarcinoma (invasive) | 3/3 | + / + + | Focal, mainly cytoplasmic reaction with little membrane positivity. Many tumour cells negative. Stroma + / + + |
| Skin | Basal cell carcinoma | 0/1 | | |
| | Malignant melanoma | 0/5 | | |
| Soft tissue | Fibrosarcoma | 1/1 | + | |

**Table II**—*continued*

| *Tissue/organ* | *Morphology* | *Number positive* | *Intensity of reaction* | *Distribution/comments* |
|---|---|---|---|---|
| Stomach | Adenocarcinomas (invasive) | 6/7 | +/++ | Membrane and cytoplasm of tumour cells positive. Variable reaction of non-neoplastic gastric mucosa –/++. Extracellular mucin positive in two cases. Stromal elements –/++. Rarely cells in lamina propria positive. |
| Testis | Seminoma | 1/5 | +/++ | Focal reaction of tumour cells in one case with syncytiotrophoblast giant cells and embryonal carcinoma faintly positive |
| Testis | Mature cystic teratoma (in testis) | 3/4 | +/++ | Focal reaction of basal layer of stratified squamous epithelium and columnar epithelium. Mucin mesenchyme weakly positive |
| | Mature cystic teratoma (metastatic from testis in lung) | 0/3 | | |
| | Anaplastic germ cell tumour including metastases × 3, MTI × 3 (one is metastatic) | 7/7 | +/+++ | Trophoblast +++, embryonal carcinoma/yolk-sac tumour +. Undifferentiated tumour ++. The reactivity is variable in some tumours many negative cells |
| Thyroid | Adenocarcinoma (metastatic to thyroid) | 0/1 | | |
| Trophoblast | Choriocarcinoma (× 4 in uterus, × 1 in lung, × 2 in brain) | 7/7 | +/+++ | Syncytiotrophoblast ++/+++, Cytotrophoblast +/++ (3 cases) show membrane and cytoplasmic reactivity |
| | Placental site trophoblastic tumour | 2/2 | ++/+++ | Most tumour cells positive (membrane predominantly) |
| | Hydatidiform mole | 1/1 | +/+++ | Syncytiotrophoblast +++ (membrane) Cytotrophoblast faintly positive. Stroma of chorionic villi negative |

The stroma of some tumours showed weak and focal reactions. This was also noted in the endothelium lining some small blood vessels in many tissues and tumours (see fetal colon, Figure 1d).

The cellular location of binding with mAb 5T4 in tumours is either membranous or cytoplasmic or a combination of both. Heavy membrane-bound location is a particular feature of syncytiotrophoblast. Cytoplasmic reactivity was predominant in pancreatic carcinomas. In gastric and breast carcinomas, both types of pattern were present. MAb 5T4 was unreactive with fixed and paraffin wax embedded tissue sections of villous trophoblast of term placentae.

*Radioimmunoassay*

Figure 4 illustrates an example of an assay for soluble antigen in human serum. $^{125}$I-labelled purified 5T4 mAb is bound to microtitre plates with bound solubilised StMPM and competed by the latter or serum. The sensitivity of this assay for 5T4 antigen is 0.1 ng ml$^{-1}$ (see Discussion). No significant 5T4 antigen was detected in serum diluted 1/100 compared with normal goat serum similarly diluted. This assay establishes that the 5T4 antigen in normal serum must be less than 10 ng ml$^{-1}$. Assays on seven normal non-pregnant sera, five first trimester, eight third trimester and eight post-partum sera gave similar results.

### Discussion

Mab 5T4 gives reactions in trophoblast which are similar to other antitrophoblast antibodies. However, our detailed immunohistochemical and previous biochemical analysis (Hole & Stern, 1988) shows that the antigen recognised is novel and distinct from the $\beta$ subunit of HCG or recently reported urinary gonadotrophin peptide (UGP) (Kardana *et al.*, 1988), PLAP (Johnson, 1984), trophoblast-leukocyte common antigen (Stern *et al.*, 1986) and those which react with mAb 18A/C4 and 18B/A5 (Loke *et al.*, 1986). Some of

**Figure 4** Radiobinding of $^{125}$I-labelled 5T4 monoclonal antibody to StMPM membrane protein bound to microtitre plates. Doubling dilutions of either normal human or goat serum with (▲, ■) or without (△, □) soluble StMPM respectively. The human and goat serum was at a final initial concentration of 1/100.

**Figure 2** Avidin-biotin peroxidase staining of 5T4 mAb in cryostat sections of (**a**) squamous carcinoma of the lung ( × 96); (**b**) invasive breast carcinoma ( × 96); (**c**) adenocarcinoma of the endometrium ( × 240) and (**d**) squamous carcinoma of the cervix ( × 96).

**Figure 3** Avidin-biotin peroxidase staining of 5T4 mAb in cryostat sections of (**a**) complete hydatidiform mole ( × 60); (**b**) choriocarcinoma showing strong labelling of syncytiotrophoblast membrane ( × 96) and (**c**) placental site trophoblast tumour showing strong membrane labelling and faint cytoplasmic reactivity ( × 150).

the differentiating immunohistochemical features are summarised below.

In contrast to antibodies directed against HCG and UGP (Kardana *et al.*, 1988) 5T4 antibody gives intense reactions with syncytiotrophoblast of term placenta. Seminomas, usually positive with mAbs against the Nalgao isozyme of PLAP (e.g. H17/E2, (Epenetos *et al.*, 1984), were almost all negative using mAb 5T4. The one case that showed some positivity was a seminoma containing syncytiotrophoblast giant cells, admixed with embryonal carcinoma. In contrast to mAbs reactive with the Regan isoenzyme of PLAP (Johnson, 1984) 5T4 mAb was usually negative with bronchiolar epithelium. Unlike both 18A/C4 and 18B/A5 antibodies mAb 5T4 did not react with villous cytotrophoblast (Loke *et al.*, 1986).

From the immunohistochemical profile, it appears that 5T4 mAb recognises an antigen with a restricted normal tissue distribution, but is reactive with a wide range of tumours. Our preliminary studies indicate that there is probably less than 10 ng $ml^{-1}$ of 5T4 antigen in normal or pregnant human serum. This is based on the fact that antigen was detectable in 1 μg $ml^{-1}$ of StMPM and that 5T4 antigen purified to homogeneity represents at most 0.001% of the protein in N-40-solubilised StMPM. The purification used WGA and 5T4 affinity chromatography with 10,000 enrichment and up to 70% yield (Hole & Stern, 1990). Thus, the assay could detect at least 0.1 ng of 5T4 $ml^{-1}$. Since the highest concentration of serum which showed acceptable non-specific inhibition was a $10^{-2}$ dilution, the upper limit for antigen is 10 ng $ml^{-1}$. These results contrast with changes in expression of PLAP detected, during pregnancy with increasing concentrations to a level of μg $ml^{-1}$ at term (Johnson, 1984) and in patients with either ovarian or testicular cancer or trophoblastic disease (Epenetos *et al.*, 1985; McLaughlin *et al.*, 1983). In spite of the presence of circulatory antigen, some antibodies have been successfully used in radioimaging of tumours (Baldwin & Byen, 1987). However, other antibodies have shown poor efficacy in uptake *in vivo* with circulating antigen present (Martin & Halpern, 1986; Pedley *et al.*, 1988). Therefore, detection of a membrane antigen may offer significant advantages in radioimmunoscintigraphy and therapy. The development of a solid phase capture assay to achieve higher levels of sensitivity comparable with PLAP assays (McLaughlin *et al.*, 1983) (0.07 ng $ml^{-1}$) will be necessary to measure the precise levels of antigen in serum from patients with different malignancies. The antibody is currently being assessed for targeting in selected patients.

We thank the Cancer Research Campaign for support, Alison McMain for technical assistance, Professor P.M. Johnson for kindly donating the pregnancy serum and Dr Elizabeth Rhodes for testing mAb in cultures of human pluripotent haematopoietic colonies. Photography was the excellent work of Mr R. Barnett.

### References

BALDWIN, R.W. & BYERS, V.S. (1987). Monoclonal antibody targeting of cytotoxic agents for cancer therapy. In *Immunology of Malignant Diseases*, Byers, U.S. & Baldwin, R.W. (eds) p. 44. MTP Press: Lancaster.

EPENETOS, A.A., TRAVERS, P., GATTER, K.G., OLIVER, R.D.T., MASON, D.Y. & BODMER, W.F. (1984). An immunological study of testicular germ cell tumours using two different monoconal antibodies against placental alkaline phosphatase. *Br. J. Cancer*, **49**, 11.

EPENETOS, A.A., SNOOK, B., HOOKER, G. & 5 others (1985). Indium-III labelled monoclonal antibody to PLAP in the detection of neoplasms of testis, ovary and cervix. *Lancet*, **ii**, 350.

HOLE, N. & STERN, P.L. (1988). A 72 kD trophoblast glycoprotein defined by a monoclonal antibody. *Br. J. Cancer*, **57**, 239.

HOLE, N. & STERN, P.L. (1990). Isolation and characterisation of 5T4, a tumour associated antigen. *Int. J. Cancer* (in the press).

JOHNSON, P.M. (1984). Immunobiology of human trophoblast. In *Immunological Aspects of Reproduction in Mammals*, Creighton, D.B. (ed) p. 109. Butterworths: London.

KARDANA, A., TAYLOR, M.E., SOUTHALL, P.J., BOXER, G.H., ROWAN, A.J. & BAGSHAWE, K.D. (1988). Urinary gonadotrophin peptides - isolation and purification, and its immunohistochemical distribution in normal and neoplatic tissues. *Br. J. Cancer*, **58**, 281.

LOKE, Y.W., BUTTERWORTH, B.H., MARGETTS, J.J. & BURLAND, K. (1986). Identification of cytotrophoblast colonies in cultures of human placental cells using monoclonal antibodies. *Placenta*, **7**, 221.

McLAUGHLIN, P.J., CHENG, M.H., SLADE, M.B. & JOHNSON, P.M. (1982). Expression on cultured human tumour cells of placental trophoblast membrane antigens defined by monoclonal antibodies. *Int. J. Cancer*, **30**, 21.

McLAUGHLIN, P.J., GEE, H. & JOHNSON, P.M. (1983). Placental-type alkaline phosphatase in pregnancy and malignancy plasma: specific estimation using a monoclonal antibody in a solid-phase enzyme immunoassay. *Clin. Chim. Acta*, **130**, 199.

MARTIN, K.W. & HALPERN, S.E. (1986). CEA production, secretion and kinetics in Balb/c mice and a nude mouse-human tumour model. *Cancer Res.*, **44**, 5475.

PEDLEY, R.B., BODEN, J.A., GREEN, A.J., GOKA, G., KEEP, P.A. & BAGSHAWE, K.D. (1988). The effect of circulating CEA on the distribution and clearance of anti-CEA antibody in a pancreatic tumour xenograft model. Third International Conference on Monoclonal Antibody Immunoconjugates for Cancer. p. 83, no. 56 (abstract).

STERN, P.L., BERESFORD, N., THOMPSON, S., JOHNSON, P.M., WEBB, P.D. & HOLE, N. (1986). Characterisation of the human trophoblast-leukocyte antigenic molecules defined by a monoclonal antibody. *J. Immunol.*, **137**, 1604.

VAITUKAKIS, J.L. (1979). Secretion of human chorionic gonadotropin by tumours. In *Carcino-embryonal Proteins, Vol. I*, Lehman, F.-G. (ed) p. 447. Elsevier: Amsterdam.

WELTE, K., PLATZER, E., LU, L. & 4 others (1985). Purification and biochemical characterisation of human pluripotent hematopoietic colony-stimulating factor. *Proc. Natl Acad. Sci. USA.*, **82**, 1526.